# Experimental effects of multi-dance sport training on student performance: a dual analysis of physical fitness and aesthetic skill development

**DOI:** 10.3389/fpsyg.2025.1522274

**Published:** 2025-08-14

**Authors:** Linling Yu, Yanan Gu, Lei Chen, Jianzhong Wan

**Affiliations:** ^1^Woosuk University, Jeonju, Republic of Korea; ^2^Hefei No.3 Middle School, Hefei, China

**Keywords:** sports dance training, students' comprehensive quality, athletic physical fitness, aesthetic ability, rumba and waltz

## Abstract

**Introduction:**

This study addresses growing concerns about declining physical fitness and insufficient aesthetic literacy among university students. These issues are compounded by the limitations of traditional physical education in supporting holistic development. Dance-based interventions are proposed as a potential solution to bridge this gap.

**Methods:**

A randomized controlled trial was conducted involving 90 female physical education majors, who were randomly assigned to one of three intervention groups: rumba, waltz, or yoga. Participants underwent 16 weeks of systematic training. Physical and aesthetic adaptations were evaluated using standardized physical tests and psychometric assessments.

**Results:**

All three interventions led to significant improvements in core strength, lower-body power, agility, and flexibility (*p* < 0.05). Rumba showed significantly greater improvements in vertical jump performance compared to waltz (MD = 4.572, *p* = 0.031), while waltz was superior to yoga in enhancing flexibility (MD = −3.196, *p* = 0.027). Aesthetic assessments revealed that waltz significantly enhanced kinesthetic awareness (MD = −17.660, *p* < 0.001), likely due to its rotational and spatial characteristics. Rumba yielded greater gains in aesthetic interpretation (MD = 17.290, *p* < 0.001), attributed to its expressive and articulated movements.

**Discussion:**

The findings highlight the genre-specific advantages of dance modalities in fostering both physical and aesthetic development. These results provide empirical support for incorporating dance-based training into higher education physical education curricula to promote holistic student development.

## 1 Introduction

With the rapid development of information technology and the deepening of globalization, the demand for talent in modern society has shifted from specialized to comprehensive. This transformation continually drives educational reform, making comprehensive quality education a key component of education reform (Tu et al., 2022; Yang, 2023). Comprehensive quality is generally defined as an integrated manifestation of multiple capabilities, including physical fitness, psychological resilience, cultural literacy, aesthetic awareness, and social adaptability, reflecting an individual’s level of coordinated development across various dimensions (Su and Zhang, 2024; Lu et al., 2024). Among the various pathways for enhancing comprehensive quality, sport dance stands out in higher education for its unique combination of artistic expression and physical training. It not only contributes to improving students’ physical functioning and aesthetic cognition but also effectively fosters holistic development in areas such as self-confidence, teamwork, cultural understanding, and creativity (Wang et al., 2024).

Numerous studies have explored the effects of physical dance on the overall quality of students. Physical ability reflects students’ health and fitness, while aesthetic ability indicates their perception of beauty. Physical dance training can significantly enhance both, providing a comprehensive measure of its impact on students’ overall quality (Liguori and Calella, 2025; Soronovych and Galai, 2025). However, most current research focuses on the effects of a single type of sport dance (Tóth and Lenténé, 2024; Li et al., 2024). There is a notable gap in exploring the effects of various types of sport dances on students’ overall quality. Based on this, the study aims to investigate the effects of various types of sport dance training on the overall quality of college students, focusing on athletic physical fitness and aesthetic ability. The study subjects consisted of 90 third-year female physical education majors from colleges and universities. Using a combination of questionnaire surveys, experimental methods, and data statistical analysis, we compared and analyzed the effects of various sports dance trainings on athletic physical fitness and aesthetic ability. Using a combination of questionnaire surveys, experimental methods, and data statistical analysis, the study compared and analyzed the effects of various sport dance trainings on athletes’ physical fitness and aesthetic ability. The study results provide empirical evidence for designing and implementing physical education dance courses in colleges and universities, offering theoretical guidance for the future development of physical education and dance training programs.

The organization of the study is as follows:

The first part is the introduction, which outlines the study’s background, purpose, and significance, while emphasizing the significant impact of physical dance on the overall quality of college students.

The second part is a literature review, which summarizes the relevant studies on sport dance and the effects of sport dance on the aesthetic ability of college students’ physical fitness capacity.

The third section is the research design, detailing the study population, subgroups, and methodologies employed.

The fourth part presents the results and analysis, comparing and analyzing the effects of various physical dance training programs on college students’ physical fitness and aesthetic ability.

The fifth section discusses the research findings of this study, comparing them with previous research results and providing relevant recommendations.

The sixth section, Conclusion, presents an overview of the results achieved in this study and proposes future research directions.

## 2 Literature review

### 2.1 Sports dance

Sports dance, also known as International Standard Dance or GB dance, is a performance form that combines dance art and sports competition, emphasizing both the grace and artistry of dance movements and the physical fitness and athletic skills required (Feng, 2023). Sports dance encompasses a variety of dance styles, primarily Latin dances (e.g., rumba, samba, cha-cha-cha, paso doble, and jive) and Standard dances (e.g., waltz, tango, quickstep, foxtrot, and Viennese waltz) (Pavlín et al., 2021). Dance for Sport training is a systematic and comprehensive process designed to enhance the technical level, physical fitness, and artistic performance of dancers (Yue, 2022). Feng et al. (2022) found that deep learning can effectively enhance the artistic ability of automated choreography for Dance for Sport, improving the accuracy of dance movements and boosting artistic creativity and educational practice. Cui and Guo (2022) developed an intelligent platform dedicated to training programs and integrated it with a signal processing diagnostic system, resulting in superior performance compared to traditional multimedia systems and significantly improving the training effectiveness of sport dance.

Nowadays, sports dance elective courses are offered in colleges and universities, and experts and scholars have conducted numerous studies on the effects of sports dance on college students. Jia (2021) explored the effects of sports dance on college students’ mental health using the MQVA algorithm on a radio network, and found that sports dance positively impacts college students’ mental health. Zhang et al. (2021) showed that regular participation in sports dance can significantly reduce depression scores among college students, potentially serving as a protective factor against depression for college students. Lei et al. (2023) also found that teaching sports dance in colleges and universities positively impacts college students’ mental health by reducing psychological barriers, improving social skills, and increasing psychological literacy. In addition to its effects on mental health, studies have also shown that prolonged sports dance training can significantly improve students’ immune function (Wang and Wang, 2021). This demonstrates that sports dance positively impacts students’ physical and mental health.

### 2.2 Research on the effect of physical dance on students’ physical ability

Physical fitness refers to the overall health and physical condition of students, which is usually assessed through fitness tests to understand their physical condition and develop suitable exercise programs (Lutkovskaya et al., 2021). For assessing physical fitness ability, Chen and Hsieh (2022) proposed a fuzzy evaluation model to assess individual physical fitness, with the main indexes including cardiorespiratory endurance, muscular endurance, muscular strength, and flexibility. Pramono et al. (2021) used discriminant analysis to develop a predictive model for health-related fitness status, with the main assessment indicators including age, weight, height, resting heart rate, blood glucose level, blood pressure, and maximal oxygen uptake. Colella and Monacis (2021) assessed changes in fitness levels in children and adolescents (9–14 years old) using four indices: standing long jump, solid ball throw, 10 × 5 round trip run, and 1-mile run test, and demonstrated significant improvements in single-movement strength tests among this group.

Enhancing physical fitness helps to strengthen students’ physical health, learning ability, and psychological quality, cultivate good living habits and social interaction skills, promote all-around development, and lay a solid foundation for their future work and life (Quka and Selenica, 2022). Numerous studies have demonstrated that sports dance training contributes to the improvement of students’ physical fitness. Wang’s M. H. (2023) study found that female college students who underwent specialized training in sports dance showed significant improvements in form, flexibility, and body composition, positively impacting their physical fitness. Brychuk et al. (2023) evaluated the effect of participation in a sports dance program on the physical fitness levels of 11–12 year old children and found that sports dance significantly improved their physical fitness levels, muscular strength, and motor skills. Sarpong (2022) assessed the fitness level of high school students in his study and found that sports dance training significantly improved students’ cardiorespiratory endurance and muscular strength.

### 2.3 Research on the effect of sport dance on students’ aesthetic ability

Aesthetic ability refers to a person’s capacity to perceive, understand, and appreciate beauty, encompassing not only visual beauty but also aesthetic experiences in music, literature, art, natural landscapes, and other areas (Fingerhut et al., 2021). Research has shown that aesthetic ability is influenced by various factors such as cultural background, education level, personal experience, and emotional experience. For example, Giannouli et al., (2022) pointed out in their study that biological and cognitive factors, such as age and gender, have different impacts on the ability to appreciate art. Regarding teaching, Wang Y. (2023) study showed that in senior music education, students can enhance their aesthetic perception and analytical abilities and establish a correct aesthetic outlook by appreciating excellent musical works. Chen and Halabi (2023) used an investigative strategy to explore the role of affective teaching values on students’ aesthetic ability, finding that affective teaching can significantly enhance students’ aesthetic ability and learning outcomes.

Spinul and Spinul (2023) believe that sport dance has realized the fusion of sport and art, and it is a work of art with certain aesthetic value and expressive power. Through multifaceted dance training and artistic cultivation, sports dance enables students to gradually develop comprehensive and profound aesthetic abilities as they perceive, appreciate, and create beauty, resulting in a significant enhancement of their aesthetic abilities (Vasiutiak et al., 2021). Therefore, sports dance is often regarded as an important means of aesthetic education within school education. Weng et al. (2021) explored the aesthetic training methods in sports dance teaching in colleges and universities and proposed measures to enhance students’ aesthetic ability through the introduction of artistic elements. Wang and Li (2023) combined theory and experiments and found that applying an aesthetic perspective in dance teaching could significantly enhance students’ aesthetic satisfaction and aesthetic ability. Guo’s (2022) study pointed out that dance teachers should, by clarifying teaching objectives and reforming teaching methods, help students grasp the style and essence of dance works, thereby enhancing students’ aesthetic ability.

### 2.4 Theoretical mechanisms of different dance styles

Different types of sport dance exhibit distinct training mechanisms in relation to physical fitness development and aesthetic enhancement, due to variations in movement structures, bodily engagement modes, and aesthetic pathways. Clarifying the technical features and psychological mechanisms of each dance genre provides a robust theoretical foundation for understanding their differential intervention effects.

As a representative of Latin dance, rumba emphasizes rhythmic transitions and emotional expressiveness. Its movement composition features frequent knee bends, hip rotations, and wave-like body motions, which intensively activate core lower limb muscle groups such as the gluteus maximus and hamstrings, thereby significantly enhancing students’ explosive leg strength and overall coordination (Wu, 2024). Moreover, rumba encourages the internalization of emotional tension through bodily articulation, allowing students to engage deeply with the fusion of rhythm and movement control—thus promoting the development of aesthetic appreciation and expressive capacity (Frías, 2023). Studies have shown that training in Latin-style dances can effectively stimulate learners’ sensitivity to artistic styles, rhythmic dynamics, and emotional expression, fostering the rapid advancement of aesthetic judgment within a relatively short timeframe (Joung and Kim, 2023).

In contrast, waltz, a core genre of Standard dance, is known for its rotational progression, spatial expansion, and trunk control. The structure of its steps requires dancers to maintain graceful coordination with the melody while executing precise control over spatial lines and movement pathways (Kuliś and Gajewski, 2022). Students must integrate core stability and lower limb propulsion to achieve seamless flow in their steps, placing higher demands on flexibility and balance. The continuity of rhythm and the coordination of posture in waltz further enhance students’ melodic perception and spatial compositional awareness, thereby activating their aesthetic receptivity (Kinoe and Sato, 2022). Some studies have also indicated that Standard dance training can significantly improve students’ integrated experiences of rhythm, musical structure, and spatial aesthetics (Fukuhara et al., 2023).

Yoga, by contrast, represents a static form of physical training. It emphasizes breathing rhythm, joint flexibility, and muscular stretching, focusing on bodily awareness and psychological relaxation during execution (Siedlaczek-Szwed and Jałowiecka-Frania, 2022). Core poses such as seated forward folds, downward dog, and spinal twists continuously stimulate the posterior chain musculature and enhance spinal flexibility, leading to steady improvements in flexibility and motor control (Rathore et al., 2024). Although yoga lacks the spatial composition and performative expressiveness inherent in traditional sport dances, its internal rhythm regulation and attention to bodily detail can indirectly evoke students’ aesthetic awareness, particularly by nurturing perceptual dimensions of aesthetic experience (Baird, 2022).

In summary, rumba emphasizes expressive contrast and rhythmic articulation, making it particularly suitable for enhancing lower limb strength and aesthetic judgment. Waltz, with its flowing rotations and spatial configurations, guides students to perceive structural beauty in movement, supporting improvements in flexibility and aesthetic sensibility. Yoga, through static stretching and somatic awareness, facilitates bodily control and foundational aesthetic engagement. These theoretical mechanisms offer strong support for the differentiated outcomes observed in the intervention effects of various dance styles.

## 3 Study design

### 3.1 Study population and subgroups

To ensure sufficient statistical power, *a priori* power analysis was conducted using G*Power 3.1. With a significance level set at *α* = 0.05, an effect size of *f* = 0.25 (medium effect), and a statistical power of 1–*β* = 0.80, the required sample size was estimated based on a repeated-measures ANOVA model (group × time interaction). Considering potential attrition and invalid data, the final sample size was determined to be 90 participants, who were randomly assigned into three groups—Experimental Group 1, Experimental Group 2, and the Control Group—each consisting of 30 participants.

The study participants were junior female students majoring in physical education at a university. To ensure group homogeneity and the validity of the intervention, the following inclusion and exclusion criteria were applied. Inclusion criteria were: (1) current enrollment as a third-year female student in a physical education program; (2) aged between 20 and 22, in good physical health, and capable of safely participating in moderate-intensity physical activity; (3) no prior systematic experience in dance training to eliminate potential confounding from pre-existing skills; and (4) provision of informed consent and willingness to complete the entire study protocol. Exclusion criteria included: (1) diagnosed conditions affecting the musculoskeletal, cardiovascular, or respiratory systems, or any health issues compromising exercise capacity; (2) participation in other physical training programs or intensive sports activities during the intervention period; and (3) psychological health concerns or poor compliance that could hinder completion of the study. All participants signed informed consent forms and explicitly confirmed that they had no prior dance training experience, thereby minimizing potential interference from prior exposure. This study received approval from the Ethics Committee on September 11, 2023 (Approval No: WSU-IRB-2023-056) and complies with ethical standards for research involving human participants.

Experimental Group 1 received rumba training, while Experimental Group 2 underwent waltz training. The control group participated in yoga training. The choice of rumba and waltz represents typical forms of Latin and Standard dance, respectively, characterized by distinct rhythmic patterns, expressive qualities, and movement styles. Yoga was selected for the control condition to ensure a basic level of physical activity while eliminating the influence of a completely sedentary lifestyle, thus enhancing intergroup comparability. Although yoga involves physical posture control and some rhythm awareness, its primary focus on breathing, flexibility, and meditation lacks the rhythmic, expressive, and spatial-aesthetic elements unique to sport dance. Hence, it was considered a low-aesthetic-load form of physical activity for this study.

To reduce external physical activity interference, participants were advised to refrain from engaging in other high-intensity or thematically related exercise programs during the intervention period. Activity compliance was monitored through self-report questionnaires and activity logs, ensuring consistency and relative independence of the intervention. All participants completed the full course of training and assessments as scheduled, with no dropouts and no missing data. Therefore, a complete dataset was used in the analysis, and no imputation or missing data handling procedures were required.

### 3.2 Experimental methods

#### 3.2.1 Questionnaire method

In this study, we designed a questionnaire based on existing scales (Dan et al., 2021) to compare the changes in aesthetic ability before and after sports dance training using three indicators: sports aesthetic receptivity, sports aesthetic appreciation, and sports aesthetic creativity. The indicators are shown in Table 1. The questionnaire options were “completely disagree,” “disagree,” “neutral,” “relatively agree,” and “strongly agree.” According to the respondents’ answers, a score of 1–5 was assigned, and the average score of each question in each indicator was taken as the final score for that indicator. A reliability test was conducted before the questionnaire was distributed to ensure the reliability and validity of the data obtained. Ninety questionnaires were distributed before and after the training, all of which were administered face-to-face. The subjects were instructed to fill in the questionnaires according to the requirements, and the final recovery rate of the questionnaires was 100%.

**Table 1:** Indicators of aesthetic ability testing.

Level 1 indicators	Secondary indicators	Questionnaire items
Esthetic ability	Esthetic sensibility	I agree that aesthetic education plays an important role in personal development.
		I agree that ‘beauty’ and ‘art’ are concepts I can understand and appreciate.
		I am willing to actively participate in sports or dance-related aesthetic activities in my spare time.
		I agree that watching sports competitions or dance performances enhances my aesthetic experience.
		I agree that I rarely experience beauty during sports dance training.* (reverse coded)
	Sports appreciation	I agree that the strength, flexibility, and rhythm in sports dance have artistic value.
		I am often moved by the perseverance and focus of sports dancers.
		I believe that improving aesthetic ability is beneficial for my future life and career development.* (reverse coded)
		I support integrating more aesthetic education into physical education curricula.
		I agree that costumes, movements, and spirit in sports dance performances all convey beauty.
	Sports creativity	I agree that participating in sports or dance performances helps improve my artistic creativity.
		I often feel the urge to dance or exercise when I hear music I like.
		I participate in sports dance training because I want to express my own sense of beauty through movement.
		I agree that sports dance training helps me regulate my physical and emotional states.
		I am willing to work or take a part-time job related to sports or dance in the future.

*indicates that the item is reverse coded, used for scoring direction control.

#### 3.2.2 Experimental methods

Before the experiment, personal information was collected from the subjects in the three groups, and a physical fitness test was conducted simultaneously. The testing indexes are shown in Table 2. In the formal experiment, each group underwent 16 weeks of sports dance training, with two sessions per week, each session lasting 45 min. The intervention duration was determined with reference to the typical instructional period of physical education courses in Chinese universities and was further informed by commonly adopted durations in sport dance intervention studies (ranging from 12 to 20 weeks). This ensured that the program allowed for observable changes in both physical fitness and aesthetic ability within a practical and evidence-based timeframe. Prior to each training or assessment session, a standardized 5-min dynamic warm-up was conducted. This included light jogging, shoulder and knee rotations, joint mobilization, and rhythm-based movement activities designed to activate core muscle groups, enhance neuromuscular readiness, and reduce the risk of injury. Physical fitness assessments were conducted indoors in a gymnasium, with the following sequence: 1-min push-ups, 1-min curl-ups, standing long jump, 50-meter sprint, sit-and-reach test, and 1-min rope skipping. A 2-min rest interval was provided between each test to allow for adequate recovery and to minimize fatigue-related interference with performance.

**Table 2:** Indicators of athletes’ physical fitness testing.

Type of quality	Assessment of indicators
Upper body strength	1 min push-up
Abdominal strength	1 min curl up
Lower extremity explosiveness	Standing long jump
Quality of speed	50-meter dash
Flexibility	Sit-up-and-bend (physical exercise)
Sensitivity	1 min jump rope

To standardize training intensity and intervention pacing, the research team developed a unified instructional management protocol prior to the study. This protocol covered training frequency, session duration, movement execution tempo, class structure, and feedback mechanisms. To quantitatively regulate and monitor training intensity, thereby ensuring the scientific rigor and replicability of the intervention process, the study established the following key control parameters and execution standards:

(1) Target Heart Rate Zone Definition: The target heart rate (THR) for participants was calculated using the Karvonen formula (Equation 1), with all three intervention groups maintained within 60–80% of their maximum heart rate to ensure moderate-intensity aerobic exercise while safeguarding participant safety.


(1) $$\mathrm{THR}=({\mathrm{HR}}_{\max}-{\mathrm{HR}}_{\text{rest}})\times \text{Intensity}+{\mathrm{HR}}_{\text{rest}}$$

Where THR refers to the target heart rate during exercise, HR_max_denotes the maximum heart rate, HR_res_ indicates the resting heart rate, and Intensity represents the desired training intensity ratio.

(2) Heart Rate Sampling Frequency and Method: All participants were equipped with Polar H10 heart rate monitors (Polar Electro, Finland), which offer high-precision real-time monitoring and recording capabilities. Heart rate data were manually recorded at three time points during each session—at the start, midpoint (25th min), and end of training. Instructors from each group also performed on-site spot checks to verify that participants maintained heart rates within the designated target zone.

(3) Movement Rhythm and Standardized Tempo Settings: Each dance style was assigned a standardized rhythm control scheme: rumba followed a 4/4 time signature at 24–27 measures per minute; waltz adhered to a 3/4 time signature at 28–30 measures per minute; yoga employed a deep breathing rhythm at 6–8 cycles per minute. All classes incorporated both metronomes and music to ensure rhythmic consistency in movement execution.

(4) Use of Rate of Perceived Exertion (RPE): At the conclusion of each session, participants were guided to self-assess exertion levels using the Borg 6–20 RPE scale. The average RPE score was maintained between 12 and 14 (“somewhat hard”), serving as a subjective validation of whether the training intensity matched the intended moderate load.

(5) Monitoring Equipment and Recording Protocols: The monitoring toolkit included the Polar H10 heart rate monitor (for heart rate), pedometers (for physical activity volume), metronomes (for rhythm regulation), and instructional logs (for session content and intensity tracking). All training data were uploaded weekly to a centralized research platform for verification and quality control. Any outliers were either excluded or re-evaluated through secondary inspection.

Although the three intervention groups differed in movement content, they were equivalent in terms of total training time, intensity control range, and organizational flow. Intervention consistency was monitored through regular on-site inspections, verification of training logs, and periodic heart rate sampling, ensuring comparability in instructional load across groups. Due to the open nature of the physical training sessions, a strict double-blind design was not feasible. However, bias control strategies were implemented during the assessment phases: physical fitness testing and questionnaire scoring were conducted by research personnel who had no involvement in the intervention implementation, thereby minimizing the risk of subjective bias in data evaluation.

Each group was instructed by a nationally certified coach specializing in either sport dance or yoga. All instructors underwent pre-intervention training focused on unified instructional procedures, including class pacing, feedback delivery style, movement standardization, and lesson plan structure. To ensure instructional consistency, all three instructors signed an implementation agreement and were subject to periodic supervision and evaluation by the research team throughout the training period. Despite differences in instructional content, the teaching format and evaluation criteria were aligned across groups to reduce potential bias stemming from instructor variability.

#### 3.2.3 Statistical methods

The experimental data were statistically analyzed using SPSS 27.0. Repeated measures ANOVA and one-way ANOVA were used to compare the post-training physical fitness test scores between groups. A *p*-value greater than 0.05 indicates that the data are not statistically significant, a *p*-value less than 0.05 indicates that the data are different, a *p*-value less than 0.01 indicates a significant difference, and a *p*-value less than 0.001 indicates a highly significant difference. To control for the accumulation of Type I error resulting from multiple comparisons, the Bonferroni correction method was applied to adjust the *p*-values in both between-group and within-group multiple comparisons. All *p*-values reported in this study reflect the adjusted results following this correction procedure.

## 4 Findings and analysis

### 4.1 Reliability and validity testing of the questionnaire

To ensure the measurement validity and applicability of the questionnaire, a pilot study was conducted using a small sample (*n* = 20) prior to the formal survey. Structural validity was assessed, and the results shown in Table 3 indicated a Kaiser–Meyer–Olkin (KMO) value of 0.812, with Bartlett’s test of sphericity yielding a significant result (*χ*^2^ = 356.241, df = 66, *p* < 0.001), confirming the questionnaire’s good structural validity. Based on this, internal consistency reliability was evaluated for each dimension of the questionnaire. As shown in Table 4, the Cronbach’s *α* coefficients for aesthetic sensibility, aesthetic appreciation, and aesthetic creativity were 0.842, 0.861, and 0.879, respectively. The overall Cronbach’s *α* coefficient of the questionnaire was 0.894. All values exceeded the threshold of 0.8, indicating a high level of reliability and demonstrating that the questionnaire yields stable and consistent measurement results.

**Table 3:** Structural validity testing results.

Test index	Value
KMO value	0.812
Bartlett’s test of sphericity	*χ*^2^ = 356.241, df = 66, *p* < 0.001

**Table 4:** Reliability testing results.

Dimension name	Aesthetic sensibility	Aesthetic appreciation	Aesthetic creativity	Total questionnaire
Cronbach’s *α*	0.842	0.861	0.879	0.894

### 4.2 Homogeneity test of physical quality and aesthetic ability of different groups

The physical quality indicators and aesthetic ability indicators of the three groups of subjects were tested for homogeneity before the experiment to determine whether the indicators of each group were at the same level. The test results are shown in Table 5. As shown in the table, the *p*-value of each index is greater than 0.05, indicating that none of the differences are statistically significant. This suggests that the physical fitness and aesthetic ability of the three groups of subjects before the experiment were at the same level, ensuring the feasibility of the subsequent experiments and the accuracy of the experimental results.

**Table 5:** Results of homogeneity test for each indicator.

Test category	Test metrics	Mean ± SD	*F*	*p*
Physical fitness of athletes	1 min push-up (reps)	25.8 ± 3.2	0.068	0.849
	1 min curl up (reps)	44.5 ± 3.8	0.094	0.711
	Standing long jump (cm)	182.6 ± 5.5	2.006	0.225
	50-meter dash (s)	7.52 ± 0.28	0.323	0.724
	Sit-up-and-bend (cm)	23.3 ± 2.6	1.129	0.337
	1 min jump rope (reps)	182.4 ± 7.2	0.217	0.553
Esthetic ability	Esthetic sensibility (score)	21.3 ± 3.2	1.361	0.229
	Aesthetic appreciation in sports (score)	23.7 ± 2.9	2.390	0.215
	Aesthetic creativity in sports (score)	22.1 ± 3.1	0.156	0.803

### 4.3 Comparative analysis of physical fitness test results of different groups of athletes

At the end of the training, a repeated measures ANOVA was performed on the test results of each index based on group (Experimental Group 1, Experimental Group 2, and control group) and time (pre- and post-experiment). The results of the analysis are shown in Table 6. As can be seen, the *p*-values for the 1-min push-up test and the 50-meter run were all greater than 0.05. This indicates that there was no statistically significant effect of time, group, or the interaction between the two on the subjects’ performance in these tests. Therefore, physical dance training did not significantly improve the subjects’ upper limb strength or speed. In the 1-min curl-up and 1-min rope skipping tests, time had a highly significant effect on the subjects’ performance in both tests, with *p*-values of less than 0.001. However, there was no statistically significant effect of grouping or the interaction of time and grouping on the performance in both tests (*p* > 0.05). This indicates that students, regardless of the type of physical dance training they received, showed statistically significant improvements in the 1-min curl-up and 1-min rope skipping tests. This resulted in a significant increase in their upper limb strength and speed. The 1-min curl-up and 1-min rope skipping scores improved significantly, indicating that physical dance training can significantly enhance students’ abdominal strength and agility.

**Table 6:** Repeated-measures ANOVA results of the test results for each indicator of physical ability.

Test metrics	Effect type	df	*F*	*p*	*η* ^2^ *p*
1 min push-up	Timing	1	0.437	0.604	0.006
	Clusters	2	0.019	0.931	0.000
	Grouping × time	2	0.002	0.893	0.000
1 min curl up	Timing	1	21.476	0.000^***^	0.163
	Clusters	2	0.641	0.597	0.026
	Grouping × time	2	0.455	0.607	0.015
Standing long jump	Timing	1	52.651	0.000^***^	0.520
	Clusters	2	0.486	0.625	0.023
	Grouping × time	2	15.649	0.000^***^	0.309
50-meter dash	Timing	1	0.245	0.611	0.008
	Clusters	2	0.476	0.605	0.033
	Grouping × time	2	0.003	0.997	0.000
Sit-up-and-bend (physical exercise)	Timing	1	7.131	0.022^*^	0.129
	Clusters	2	1.037	0.506	0.047
	Grouping × time	2	4.392	0.26^*^	0.043
1 min jump rope	Timing	1	18.217	0.000^***^	0.185
	Clusters	2	0.366	0.526	0.013
	Grouping × time	2	0.715	0.420	0.026

* and *** indicate *p* < 0.05, and *p* < 0.001, respectively.

In the standing long jump test, both time and the interaction of time and grouping had a highly significant effect on performance (*p* < 0.001), whereas grouping alone had no statistical effect on performance. This indicates that students’ standing long jump performance could be significantly improved after physical dance training, with some variations depending on the type of physical dance training. Therefore, further multiple comparisons of the test results were conducted, and the results are presented in Table 7. The Mean Difference (MD) is used to represent the difference in means between groups or time points and serves as a direct indicator for evaluating the effectiveness of the training intervention. As shown by the results, after 16 weeks of sport dance training, a significant difference was observed between Experimental Group 1 and Experimental Group 2 in the post-intervention standing long jump performance (MD = 4.572, *p* = 0.031, *d* = 1.016), indicating that rumba training had a distinct advantage in enhancing lower limb explosive power. This effect may be attributed to the frequent knee bends, hip rotations, and push steps characteristic of rumba, which consistently activate core lower limb muscle groups. The explosive engagement of the quadriceps, gluteus maximus, and hamstrings likely contributes to the strength-oriented loading nature of the training. In contrast, no significant difference was found between Experimental Group 2 and the Control Group (MD = 0.129, *p* = 1.020), suggesting that the improvement in lower limb explosive strength from waltz training was comparable to that of yoga. Given that waltz involves predominantly slow, controlled movements emphasizing gliding and balance, it tends to target gait coordination rather than explosive power, which may explain the lack of significant gains in jump performance.

**Table 7:** Multiple comparisons of standing long jump test results.

Between-Group comparison	Group comparison (G1–G2)	MD (G1–G2)	SE	95% CI	*p*	Cohen’s *d*
Post-training	Experimental Group 1 vs. Group 2	4.572	1.519	[1.595, 7.549]	0.031^*^	1.016
	Experimental Group 2 vs. Control	0.129	1.693	[−3.189, 3.447]	1.020	0.027
	Control vs. Experimental Group 1	−4.365	0.025	[−7.760, −0.970]	0.025^*^	0.949

Within-Group comparison	Time comparison (T1–T2)	MD (T1–T2)	SE	95% CI	*p*	Cohen’s *d*
Experimental Group 1	Pre-post	−8.476	1.138	[−10.706, −6.246]	0.000^***^	1.256
Experimental Group 2	Pre-post	−2.664	1.109	[−4.838, −0.490]	0.033^*^	0.507
Control Group	Pre-post	−1.922	0.952	[−3.788, −0.056]	0.073	0.384

All *p*-values reported above have been adjusted using the Bonferroni correction method. The same applies to subsequent tables. MD¸ mean difference; positive values indicate Group 1 > Group 2; negative values indicate Group 1 < Group 2. * and *** indicate *p* < 0.05, and *p* < 0.001, respectively.

A significant difference was also observed between Experimental Group 1 and the Control Group (MD = −4.365, *p* = 0.025), further confirming the unique effectiveness of rumba in improving explosive lower limb strength. In contrast, yoga training mainly consists of static stretching and flexibility control, with limited stimulation of fast-twitch muscle fibers, and therefore has minimal impact on explosive performance measures. In terms of within-group longitudinal comparisons, Experimental Group 1 showed a highly significant improvement before and after training (MD = −8.476, *p* = 0.000, *d* = 1.256), corresponding to a large effect size. This further highlights the systematic and targeted effect of rumba training on explosive power enhancement. While Experimental Group 2 also achieved a statistically significant improvement (MD = −2.664, *p* = 0.033, *d* = 0.507), the effect size was moderate, indicating a relatively milder impact. The Control Group exhibited a slight, non-significant increase in performance (MD = −1.922, *p* = 0.073, *d* = 0.384), suggesting that participants without specialized training are unlikely to achieve meaningful improvements in lower limb explosiveness within a short time frame.

As shown in the results of Table 8, the performance in the sit-and-reach test was significantly influenced by both time and the interaction between time and group (*p* < 0.05), while the main effect of group was not significant. This suggests that sport dance training, in general, effectively improves students’ flexibility, and that the degree of improvement varies across different dance styles. Between-group comparisons revealed that Experimental Group 2 performed significantly better than the Control Group after training (MD = −3.196, *p* = 0.027, *d* = 0.657), indicating a clear advantage of waltz training in enhancing flexibility. This effect may be attributed to the high demands of waltz on trunk control, expansive step execution, and overall bodily coordination. Particularly, the continuous engagement of the abdominal and posterior thigh muscles during step extension and core traction movements likely contributed to the improved trunk flexion required in the sit-and-reach task. In contrast, no significant difference was found between Experimental Group 1 and Experimental Group 2 (*p* = 0.672), nor between Experimental Group 1 and the Control Group (*p* = 0.381). Although rumba incorporates elements of hip control and movement extension, its training emphasis is primarily on weight shifting and lower limb force production. As a result, the stimulus it provides to flexibility development appears to be limited, failing to produce a significant advantage in this specific test.

**Table 8:** Multiple comparisons of the results of the seated forward bending test.

Between-Group comparison	Group comparison (G1–G2)	MD (G1–G2)	SE	95% CI	*p*	Cohen’s *d*
Post-training	Experimental Group 1 vs. Group 2	1.174	1.026	[−0.837, 3.185]	0.672	0.651
	Experimental Group 2 vs. Control	−3.196	1.268	[−5.681, −0.711]	0.027^*^	0.657
	Control vs. Experimental Group 1	1.747	1.059	[−0.329, 3.823]	0.381	1.251

Within-Group comparison	Time comparison (T1–T2)	MD (T1–T2)	SE	95% CI	*p*	Cohen’s *d*
Experimental Group 1	Pre-post	−0.512	1.204	[−2.872, 1.848]	0.824	1.304
Experimental Group 2	Pre-post	−0.580	1.109	[−2.754, 1.594]	0.715	0.983
Control Group	Pre-post	−4.528	0.976	[−6.441, −2.615]	0.000^***^	0.326

MD, mean difference; positive values indicate Group 1 > Group 2; negative values indicate Group 1 < Group 2. * and *** indicate *p* < 0.05, and *p* < 0.001, respectively.

Regarding within-group comparisons, only the Control Group showed a significant pre- to post-intervention improvement (MD = −4.528, *p* = 0.000, *d* = 0.326), indicating that yoga training consistently promoted students’ flexibility. Yoga focuses on static stretching and joint range control, with movements such as seated forward folds and spinal extensions providing sustained stimulation to the hamstrings and lumbar musculature, directly enhancing trunk mobility and posterior chain flexibility. In comparison, Experimental Group 1 (MD = −0.512, *p* = 0.824) and Experimental Group 2 (MD = −0.580, *p* = 0.715) did not exhibit significant pre-post changes, suggesting that while dance training may support dynamic muscular control, it may lack the intensity and continuity required to produce meaningful gains in static flexibility when compared to yoga.

### 4.4 Comparative analysis of the results of different groups of aesthetic ability tests

Aesthetic ability, as a core component of comprehensive quality, reflects not only an individual’s cognitive perception of beauty but also the multidimensional development of emotional sensitivity, artistic understanding, and creative potential. As shown in the results of Table 9, the main effect of time was highly significant across all three dimensions of aesthetic ability—aesthetic sensibility, aesthetic appreciation, and aesthetic creativity (*p* < 0.001). This indicates that regardless of the training modality, 16 weeks of sport dance or yoga intervention effectively enhanced students’ overall aesthetic competence.

**Table 9:** Repeated-measures ANOVA results of the survey results for each indicator of aesthetic ability.

Test metrics	Effect type	df	*F*	*p*	η^2^p
Esthetic sensibility	Timing	1	47.551	0.000^***^	0.377
	Clusters	2	0.783	0.366	0.042
	Grouping × time	2	6.257	0.002^**^	0.154
Esthetic appreciation	Timing	1	33.004	0.000^***^	0.441
	Clusters	2	0.377	0.700	0.022
	Grouping × time	2	4.431	0.013^*^	0.263
Esthetic creativity	Timing	1	77.635	0.000^***^	0.476
	Clusters	2	0.499	0.590	0.037
	Grouping × time	2	2.694	0.053	0.088

*, **, and *** indicate *p* < 0.05, *p* < 0.01 and *p* < 0.001, respectively.

Significant interaction effects were observed between group and time in both aesthetic sensibility (*F* = 6.257, *p* = 0.002, η^2^p = 0.154) and aesthetic appreciation (*F* = 4.431, *p* = 0.013, η^2^p = 0.263), suggesting that different types of dance training produced divergent improvement trajectories in aesthetic ability. These differences are closely related to the expressive characteristics of each dance style. Waltz, as a representative of Standard dance, is marked by elegant movement structure, flowing rhythm, and strong spatial expansiveness. Students are required to perceive and simulate melodic lines and spatial configurations during training, which offers strong guidance in enhancing aesthetic sensibility. This is further supported by the multiple comparison results, which revealed that the waltz group significantly outperformed the control group in post-training aesthetic sensibility (MD = 8.433, *p* = 0.006), with a highly significant pre-post improvement (MD = −17.660, *p* < 0.001). In contrast, the most notable enhancement in aesthetic appreciation was observed in the rumba group. Rumba is characterized by its intense rhythm, emotional tension, and expressive bodily dynamics. Its training demands deep engagement in emotional expression and refined control over movement expansion and contraction. Such embodied experiences foster students’ capacity to appreciate and evaluate the beauty of dance. Experimental Group 1 showed a highly significant improvement in aesthetic appreciation (MD = −17.290, *p* < 0.001), surpassing the gains made by both the control and Experimental Group 2, indicating that rumba more effectively facilitates the development of students’ aesthetic discernment and evaluative understanding of dance art. It is worth noting that although the main effect of time on aesthetic creativity was also highly significant (*F* = 77.635, *p* < 0.001, η^2^p = 0.476), neither the group effect nor the interaction effect reached statistical significance (*p* > 0.05). This suggests that all types of training similarly promoted students’ capacity for creative aesthetic expression, with no significant divergence between groups. Such results may be attributed to the nature of the training content, which was largely centered on imitation and structured repetition, lacking elements of improvisation, movement composition, or other advanced forms of expressive practice. These limitations may have constrained the activation of students’ creative potential in aesthetic domains. Overall, this study confirms the significant role of sport dance training in enhancing students’ aesthetic ability. It also reveals differentiated improvement pathways depending on the dance style: Standard dance (e.g., waltz) emphasizes perceptual guidance, while Latin dance (e.g., rumba) cultivates emotional comprehension and expressive ability. In contrast, the development of aesthetic creativity may require more open-ended, improvisation-driven training designs to fully stimulate students’ creative expression.

To further explore the effects of different sport dance training methods on aesthetic sensibility and aesthetic appreciation, multiple comparisons were conducted for these two indicators. Table 10 presents the multiple comparison results for aesthetic sensibility. As shown, in the post-training between-group comparisons, Experimental Group 2 scored significantly higher than the Control Group in aesthetic sensibility (MD = 8.433, *p* = 0.006, Cohen’s *d* = 1.339), indicating a notable advantage of waltz training in enhancing students’ experience and perception of the beauty inherent in sport dance. The waltz is characterized by continuous rotation and fluid movement, accompanied by gentle musical rhythms and graceful body postures. It emphasizes the interaction between the body and spatial environment. This fusion of “form and emotion” enables students to develop a direct perception of rhythm, bodily lines, and emotional flow throughout the training process, thereby significantly boosting their aesthetic sensibility. Although there was a numerical difference between Experimental Groups 1 and 2 (MD = −4.770), it did not reach statistical significance (*p* = 0.255), suggesting that while Latin and Standard dance styles have distinct characteristics, they did not produce fundamentally different effects on the enhancement of aesthetic sensibility within the given training period. Likewise, no significant difference was found between Experimental Group 1 and the Control Group (*p* = 0.442), indicating that the impact of rumba on aesthetic sensibility was relatively unstable in this study. This may be due to its emphasis on emotional tension and contrast in movement dynamics, which students may not have fully internalized during the limited duration of the intervention.

**Table 10:** Multiple comparisons of aesthetic sensibility survey results.

Between-Group comparison	Group comparison (G1–G2)	MD (G1–G2)	SE	95% CI	*p*	Cohen’s *d*
Post-training	Experimental Group 1 vs. Group 2	−4.770	2.984	[−10.619, 1.079]	0.255	0.954
	Experimental Group 2 vs. Control	8.433	2.714	[3.114, 13.752]	0.006^**^	1.339
	Control vs. Experimental Group 1	−3.725	2.833	[−9.278, 1.828]	0.442	0.621

Within-Group comparison	Time comparison (T1–T2)	MD (T1–T2)	SE	95% CI	*p*	Cohen’s *d*
Experimental Group 1	Pre-post	−7.643	2.795	[−13.121, −2.165]	0.026^*^	1.390
Experimental Group 2	Pre-post	−17.660	2.722	[−22.995, −12.325]	0.000^***^	1.463
Control Group	Pre-post	−4.873	2.599	[−9.967, 0.221]	0.031^*^	0.750

MD, mean difference; positive values indicate Group 1 > Group 2; negative values indicate Group 1 < Group 2. *, **, and *** indicate *p* < 0.05, *p* < 0.01 and *p* < 0.001, respectively.

In the within-group longitudinal comparisons, all three groups showed significant pre-post improvements. Experimental Group 2 demonstrated the most pronounced enhancement (MD = −17.660, *p* = 0.000, *d* = 1.463), representing a “very large” effect size and reinforcing the strong aesthetic educational value of waltz in the perceptual domain. Experimental Group 1 also showed a significant improvement (MD = −7.643, *p* = 0.026, *d* = 1.390), indicating that the movement structure of rumba, which emphasizes emotional expression and control of body curves, possesses substantial aesthetic appeal. Although the Control Group lacked dance-specific elements, it also exhibited a significant improvement (MD = −4.873, *p* = 0.031, *d* = 0.750), which may be attributed to yoga’s emphasis on bodily awareness, rhythm control, and meditative relaxation. These elements likely activated students’ internal perception of the body-rhythm relationship, thereby indirectly enhancing their aesthetic sensibility.

In summary, all three training modalities effectively enhanced students’ aesthetic sensibility to varying degrees. Among them, waltz training demonstrated the most prominent intervention effect, likely due to the artistic quality and rhythmic fluidity of its movements, which readily evoked aesthetic experiences in a short time frame.

As shown in Table 11, although the between-group differences in aesthetic appreciation scores did not reach statistical significance (*p* > 0.05), observable trends emerged when examining mean differences and effect sizes. Post-intervention, Experimental Group 1 scored higher than Experimental Group 2 (MD = 6.228, *p* = 0.073, *d* = 0.872), with an effect size approaching the threshold for a “large” effect. This suggests that rumba may offer a greater advantage in enhancing students’ understanding of the structural and stylistic features of dance aesthetics. Rumba emphasizes emotional expression, movement tension, and rhythmic transitions—its inherently dramatic and stylized nature compels students to develop a deeper awareness of artistic style distinctions and expressive nuances. This, in turn, fosters more refined aesthetic judgment and evaluative depth. The difference between Experimental Group 2 and the Control Group was minimal (MD = −0.380, *p* = 0.855), indicating that short-term waltz training in this study did not significantly enhance students’ structural aesthetic analysis. As a dance style centered on smoothness and balance, the aesthetic appeal of waltz lies more in perceptual flow and overall visual coherence, offering a more indirect influence on the cognitive dimension of aesthetic appreciation. Comparatively, the difference between the Control Group and Experimental Group 1 approached significance (MD = −5.931, *p* = 0.086), further supporting the potential of rumba training to stimulate students’ interest and capability in analyzing dance construction and stylistic nuances.

**Table 11:** Multiple comparisons of aesthetic appreciation survey results.

Between-Group comparison	Group comparison (G1–G2)	MD (G1–G2)	SE	95% CI	*p*	Cohen’s *d*
Post-training	Experimental Group 1 vs. Group 2	6.228	2.506	[1.316, 11.140]	0.073	0.872
	Experimental Group 2 vs. Control	−0.380	2.719	[−5.709, 4.949]	0.855	1.059
	Control vs. Experimental Group 1	−5.931	2.706	[−11.235, −0.627]	0.086	0.533

Within-Group comparison	Time comparison (T1–T2)	MD (T1–T2)	SE	95% CI	*p*	Cohen’s *d*
Experimental Group 1	Pre-post	−17.290	2.647	[−22.478, −12.102]	0.000^***^	1.242
Experimental Group 2	Pre-post	−6.296	2.573	[−11.339, −1.253]	0.028^*^	1.281
Control Group	Pre-post	−5.683	2.745	[−11.063, −0.303]	0.047^*^	0.690

MD, mean difference; positive values indicate Group 1 > Group 2; negative values indicate Group 1 < Group 2. *, and *** indicate *p* < 0.05 and *p* < 0.001, respectively.

In the within-group longitudinal comparisons, all three groups demonstrated significant improvements. Experimental Group 1 showed the most pronounced pre-post difference (MD = −17.290, *p* = 0.000, Cohen’s *d* = 1.242), reaching a “very large” effect size, indicating that rumba training was particularly effective in deepening students’ understanding and evaluation of dance structure, rhythmic design, and movement artistry. Both Experimental Group 2 and the Control Group also achieved statistically significant improvements (*p* = 0.028 and *p* = 0.047, respectively), although with slightly smaller effect sizes (*d* = 1.281 for Group 2 and *d* = 0.690 for the Control Group). The improvement in the Control Group may be attributed to yoga’s emphasis on meditative focus, posture aesthetics, and bodily awareness, which can indirectly stimulate aesthetic reflection on physical expressiveness.

Although the post-intervention differences between groups did not reach full statistical significance, the observed effect sizes and within-group improvements indicate that rumba training, through its expressive richness and artistic structure, more effectively guides students toward a systematic understanding of movement style, emotional tension, and dance form. As such, it demonstrates a stronger intervention effect on the dimension of aesthetic appreciation.

## 5 Discussion

This study focuses on junior female students majoring in physical education at colleges and universities. It combines the questionnaire survey method, experimental method, and data statistics method to investigate the influence of multi-dance sports dance training on students’ comprehensive quality from the perspectives of athletes’ physical fitness and aesthetic ability. The results of the study show that after sports dance training of different dance types, the students’ abdominal strength, lower limb explosive power, sensitivity, and flexibility were significantly improved. This indicates that sports dance training can enhance the students’ physical fitness. The effect sizes (Cohen’s *d*) associated with each training modality in improving physical fitness and aesthetic ability also demonstrate substantial practical significance. Notably, rumba training produced very large effect sizes in both lower limb explosive strength (*d* = 1.256) and aesthetic appreciation (*d* = 1.242), indicating that this dance style can induce systematic enhancement in core muscular power and artistic evaluative capacity within a relatively short intervention period, showcasing strong intervention value. From a practical perspective, these effect sizes represent substantial improvements with educational significance. Specifically, rumba training led to an average increase of approximately 8.5 cm in standing long jump performance (*d* = 1.256), which corresponds to a 4.6% improvement over the baseline level of 182.6 cm. This indicates a significant short-term enhancement in lower-body explosive strength, highlighting the clear intervention value of rumba in physical education practice. Similarly, in terms of aesthetic sensibility, the waltz group demonstrated an average improvement of nearly 17.7 points after training (*d* = 1.463), elevating the participants’ scores from a baseline of 21.3 to nearly 39. This exceeds the threshold typically considered educationally meaningful, indicating a substantial enhancement in participants’ perception of rhythm, spatial composition, and body form aesthetics—far greater than the 5–8 point gains commonly observed in general curricular interventions. Regarding flexibility, yoga training led to an average improvement of about 4.5 cm in the sit-and-reach test (*d* = 0.326), which falls within a moderate-to-high effect range. Given the baseline average of 23.3 cm, this reflects a 19.4% increase, suggesting that even in the absence of expressive dance elements, static stretching can steadily improve body control capacity. Based on differing instructional goals, universities may consider prioritizing rumba in courses aimed at integrating physical and artistic development, employing waltz in aesthetic perception modules, and incorporating yoga into flexibility and mindfulness training sessions, thereby optimizing the differentiation and effectiveness of physical-aesthetic education curricula.

While previous research provided a theoretical foundation for this study, the present research introduces several innovations in terms of experimental design, dance style selection, and variable decomposition, deepening the exploration of sport dance as a pathway to holistic student development. For example, Tunçgenç et al. (2024) found that a 5-week online hip-hop dance program significantly improved social connection and well-being among British adolescents. However, their study focused on standardized pre- and post-intervention comparisons of subjective well-being and social interaction, without differentiating between dance styles. This study not only extended the intervention to 16 weeks but also introduced rumba and waltz as representative styles of Latin and Standard dance, respectively, using a controlled design to systematically compare their differential effects on physical and aesthetic dimensions—enhancing the study’s specificity and external validity. Tung et al. (2024) examined the impact of dance interventions on children’s cognitive control, focusing on neural and attentional indicators. While valuable in offering a neurological perspective on cognitive performance, their research overlooked the potential of dance as a holistic medium for aesthetic and emotional experiences. In contrast, this study incorporated a tripartite model of aesthetic ability—sensibility, appreciation, and creativity—and developed quantifiable measures to systematically examine how different dance styles influence distinct layers of aesthetic development, thus expanding the scope of sport dance research in educational psychology. Zhang et al. (2025), in their study on the effects of dance interventions on physical balance and cognitive function in older adults, confirmed the multisystemic benefits of dance. However, their participant pool was limited to older individuals, and the training content was based on unified choreography, lacking detailed investigation into how different dance styles affect specific functional domains. By contrast, this study revealed that rumba—with its focus on emotional expression and bodily control—is more effective in enhancing aesthetic appreciation, while waltz—with its rhythmic structure and spatial design—is more beneficial for improving aesthetic sensibility and flexibility. These findings enrich the application of dance intervention strategies in youth educational settings.

Theoretically, this study contributes by comparing the differential effects of various dance styles across physical and aesthetic dimensions, thus enriching the conceptual framework for sport dance-based training. Moreover, by dividing aesthetic ability into three measurable dimensions—sensibility, appreciation, and creativity—it enhances the structural integrity and operational feasibility of aesthetic education research in sport dance. In practice, several recommendations are proposed: First, to achieve instructional goals centered on enhancing students’ lower limb explosive power and aesthetic appreciation, rumba training should be designated as a core module within university physical education curricula—particularly in quality development or expressive movement courses. Instructional focus should be placed on knee bends, hip rotations, and rhythmic transitions, supplemented by movement style analysis and imitation-based practice to deepen students’ understanding of dance structure, emotional expression, and muscular tension. Classroom demonstrations and peer feedback sessions are also recommended to facilitate the practical translation of aesthetic judgment into observable outcomes. Second, within course frameworks that emphasize the cultivation of aesthetic sensibility and body coordination, waltz is recommended as the primary instructional content. It is particularly suitable for aesthetic-oriented physical education courses or interdisciplinary art-based experiential programs in higher education. Teaching strategies should incorporate multimodal elements such as spatial composition, melodic perception, and audiovisual feedback, guiding students to experience the fluidity of movement and spatial rhythm through the fusion of posture and tempo—thereby stimulating affective cognition and artistic engagement. Third, yoga training may serve as a supplementary module for improving flexibility and bodily control, ideally scheduled in alternating sessions throughout the academic term to support physical recovery and psychological regulation. It is recommended to designate one static training session per week to establish a dynamic-static instructional rhythm. Students should also be encouraged to track and document changes in physical condition, thereby enhancing self-awareness and reflective capacity throughout the training process. Finally, to further improve the educational effectiveness of sport dance curricula, it is advisable to construct a “multi-style–multi-objective–multi-strategy” course system. This approach would involve systematically incorporating pre-post effect size indicators into curricular evaluation and developing dynamic assessment models. In day-to-day instruction, open-ended tasks such as choreography, improvisational performance, and aesthetic critique should be integrated to transition students from passive recipients to active creators—fostering the holistic development of physical-aesthetic literacy.

Despite providing robust evidence of the effects of multi-style sport dance training on students’ physical fitness and aesthetic ability, this study has certain limitations. First, the study sample consisted exclusively of third-year female students majoring in physical education at a single university, resulting in high homogeneity in terms of gender, training background, and academic standing. This lack of demographic diversity introduces a degree of population-specific response to the intervention, meaning that the observed effects primarily reflect outcomes within this specific cohort. Consequently, the findings cannot be generalized to male students, underclassmen, or individuals outside of physical education disciplines, thereby limiting the study’s external validity. Second, although the aesthetic ability was assessed using a questionnaire instrument that had undergone reliability and validity testing, the self-report nature of this tool inherently depends on participants’ subjective judgments. As such, the results are vulnerable to influences from transient emotional states, social desirability biases, and self-perception distortions, which may lead to inflated estimates of training efficacy and constrain the objectivity of aesthetic improvement assessments. Third, the study employed a single post-intervention measurement point, with no follow-up assessments conducted. This design choice precludes the evaluation of effect sustainability, delayed responses, or potential attenuation over time, leaving unanswered questions regarding the temporal stability and cumulative impact of the training. Finally, although participants were instructed to refrain from engaging in other strenuous physical activities during the intervention period, key behavioral covariates—such as dietary patterns, sleep quality, psychological condition, and extracurricular physical activity—were not systematically tracked or controlled. These uncontrolled variables may have influenced the natural fluctuations in physical fitness and aesthetic competence, thereby introducing potential confounds and diminishing the precision of causal attribution regarding the observed training effects.

## 6 Conclusion

This research integrates questionnaire surveys, experimental approaches, and statistical analysis of data to examine and compare how various sports dance training programs influence university students’ overall development, particularly focusing on their physical conditioning and aesthetic skills. Key findings include:

(1) The three physical dance training methods did not substantially enhance students’ upper-body strength or speed-related attributes (*p* > 0.05). However, moderate to significant improvements were observed in core strength, leg explosiveness, flexibility, and coordination (*p* < 0.05).
(2) Statistical analysis revealed Rumba demonstrated superior effectiveness to Waltz and Yoga in enhancing students’ standing long jump performance (MD = 4.572, *p* = 0.031). Within-group comparisons showed highly significant pre/post-training differences between rumba and waltz (*p* < 0.001), confirming both dance modalities substantially boost lower limb explosive strength.
(3) Statistical analysis showed that waltz training surpassed yoga in increasing seated forward flexion (MD = −3.196, *p* = 0.027). Meanwhile, the control group displayed a highly notable change post-intervention (*p* < 0.001), confirming that yoga also improves flexibility—just not as effectively as waltz.
(4) Statistical analyses confirmed all three training modalities (waltz, rumba, and yoga) effectively improved students’ aesthetic capabilities. Waltz demonstrated the most pronounced enhancement of aesthetic perception (MD = −17.660, *p* = 0.000), whereas rumba showed superior efficacy in developing aesthetic evaluation skills (MD = −17.290, *p* < 0.001).

This study highlights the differential impacts of various sport dance styles on students’ physical fitness and aesthetic ability. Future research should consider expanding the participant pool to include more representative populations across gender, academic disciplines, and educational stages, thereby examining the generalizability and specificity of sport dance interventions across diverse groups. Investigating the moderating role of gender in movement receptivity, aesthetic preference, and training feedback could enrich the development of personalized intervention strategies in physical-aesthetic education. Second, given the inherent subjectivity of current questionnaire-based assessments, it is recommended to incorporate more objective evaluation methods. These may include behavioral observation checklists, expert rating systems, and technology-assisted tools such as video-based motion capture and AI-driven recognition systems, which can quantitatively assess changes in movement performance, body tension control, and emotional expressiveness—thus enhancing the measurement validity of non-cognitive domains like aesthetic ability. Third, to more comprehensively capture the dynamic trajectory of training effects, future studies are encouraged to adopt longitudinal designs with distributed follow-up intervals—such as at 1 month, 3 months, and 6 months post-intervention—to examine the persistence, attenuation, or potential rebound of intervention effects across cognitive, behavioral, and emotional dimensions. Such designs would provide theoretical grounding for the long-term optimization and consolidation of intervention strategies. Finally, to mitigate the influence of potential confounding variables in the training environment, future studies may incorporate behavioral control strategies and covariate modeling approaches. By systematically tracking behavioral factors such as daily dietary intake, sleep patterns, psychological states, and exercise logs, and incorporating these variables into statistical analyses as covariates, researchers can enhance the precision of causal attribution regarding intervention effects.

## Data Availability

The original contributions presented in the study are included in the article/supplementary material, further inquiries can be directed to the corresponding author.
